# *Clostridium difficile* infection perceptions and practices: a multicenter qualitative study in South Africa

**DOI:** 10.1186/s13756-018-0425-y

**Published:** 2018-10-29

**Authors:** Laurel Legenza, Susanne Barnett, Warren Rose, Nasia Safdar, Theresa Emmerling, Keng Hee Peh, Renier Coetzee

**Affiliations:** 1 University of Wisconsin-Madison School of Pharmacy, 777 Highland Ave, Madison, WI 53705 USA; 2 University of the Western Cape School of Pharmacy, Robert Sobukwe, Cape Town, 7535 South Africa; 3 University of Wisconsin School of Medicine and Public Health, 750 Highland Ave, Madison, WI 53726 USA

**Keywords:** Healthcare associated infection, Infection control, Qualitative study, Antimicrobial stewardship, Global health

## Abstract

**Background:**

*Clostridium difficile* infection (CDI) is understudied in limited resource settings. In addition, provider awareness of CDI as a prevalent threat is unknown. An assessment of current facilitators and barriers to CDI identification, management, and prevention is needed in limited resource settings to design and evaluate quality improvement strategies to effectively minimize the risk of CDI.

**Methods:**

Our study aimed to identify CDI perceptions and practices among healthcare providers in South African secondary hospitals to identify facilitators and barriers to providing quality CDI care. Qualitative interviews (11 physicians, 11 nurses, 4 pharmacists,) and two focus groups (7 nurses, 3 pharmacists) were conducted at three district level hospitals in the Cape Town Metropole. Semi-structured interviews elicited provider perceived facilitators, barriers, and opportunities to improve clinical workflow from patient presentation through CDI (1) Identification, (2) Diagnosis, (3) Treatment, and (4) Prevention. In addition, a summary provider CDI knowledge score was calculated for each interviewee for seven components of CDI and management.

**Results:**

Major barriers identified were knowledge gaps in characteristics of *C. difficile* identification, diagnosis, treatment, and prevention. The median overall CDI knowledge score (scale 0–7) from individual interviews was 3 [interquartile range 0.25, 4.75]. Delays in *C. difficile* testing workflow were identified. Participants perceived supplies for CDI management and prevention were usually available; however, hand hygiene and use of contact precautions was inconsistent.

**Conclusions:**

Our analysis provides a detailed description of the facilitators and barriers to CDI workflow and can be utilized to design quality improvement interventions among limited resource settings.

## Background

*Clostridium difficile* infection (CDI) is an increasingly important healthcare-associated infection associated with long hospitalisations and high patient morbidity and mortality [1]. CDI often results from normal gut bacterial disruption due to broad-spectrum antimicrobial use, allowing for overgrowth of toxigenic *C. difficile*. CDI outbreaks have been reported extensively in the United States (US) and Europe over the last two decades. CDI in these hospitals is prevalent supporting extensive CDI prevention and control measures. However, CDI is understudied in low and limited resource settings, including nearly all African countries. Where limited data exists, a study at a tertiary hospital in Cape Town, South Africa found 22% of stool samples from patients with suspected CDI diarrhoea were *C. difficile* positive [2]. In addition, patients in South Africa are disproportionately affected by HIV and tuberculosis (TB) and therefore also experience known CDI risk factors of prior hospital and antibiotic exposure—exposures that can uniquely contribute to an increased risk of CDI and poor outcomes [3, 4].

Treatment of CDI requires a comprehensive approach that includes infection prevention and control (IPC) measures to limit transmission and prevent outbreaks. Although no CDI IPC guidelines exist specific to African countries, the Infectious Diseases Society of America (IDSA) and European Society of Clinical Microbiology and Infectious Diseases guidelines consistently recommend IPC components of antimicrobial stewardship programs (ASP) which include effective environment cleaning, patient isolation, use of personal protective equipment such as gowns and gloves, surveillance, and education [5]. These evidence-based recommendations are key to effective CDI management. The feasibility of using these recommendations in populations with limited healthcare resources has not been established. In addition, healthcare provider knowledge of CDI and the guidance to effectively mitigate and manage patient populations at higher risk for CDI is unknown.

Provider knowledge of CDI and treatment measures are essential to both successfully manage CDI and prevent disease transmission. An assessment of current facilitators and barriers to CDI identification, management, and prevention is needed to design and evaluate improvement strategies to effectively minimize the risk of CDI. To our knowledge, no comprehensive study of barriers and facilitators to CDI workflow (identification, diagnosis, treatment, and prevention) in Sub-Saharan Africa exists. Our study aims to fill this gap by eliciting CDI perceptions and management practices among healthcare providers in South African secondary hospitals to uncover facilitators and barriers to providing quality CDI care.

## Methods

### Data collection

We utilized a qualitative approach to elicit health care providers’ perceptions of barriers and facilitators to CDI management because it provides detailed process oriented results. We conducted semi-structured interviews and focus groups among clinical providers at three secondary hospitals in South Africa. A Systems Engineering Initiative for Patient Safety (SEIPS) model served as a framework for the interview guide. The SEIPS framework connects work systems to patient and organizational outcomes, while including interactions in the work system between available tools, people, tasks, the internal environment, and the organization [6]. The semi-structured interview assessed each subject’s CDI knowledge and traced workflow from patient presentation with CDI symptoms through CDI 1.) Identification, 2.) Diagnosis, 3.) Treatment, and 4.) Prevention. Interview questions were structured to reveal facilitators and barriers to these CDI workflow steps and opportunities to improve CDI treatment. The interview guide included optional probes to use when appropriate to gather additional information. When participants revealed a lack of CDI knowledge from the preliminary questions, the interview was then modified to contain general questions about diarrhoea management. As a qualitative study, the interviewer could use information gathered from prior interviews to direct future interview discussions and build on emerging concepts. For example, asking for further detail and implications on processes mentioned with open-ended questions.

### Participants

Providers working in three public secondary (district) level hospitals in the Western Cape, Cape Town Metropole, South Africa were invited to participate in this study. The three participating hospitals, averaging 265 inpatient beds overall, were previously selected to be included in a CDI quality improvement intervention. Our study aimed to interview, at minimum, 15 providers among five provider types including frontline nurses, nurse managers, pharmacists, junior physicians (registrars and medical officers), and senior physicians (consultants and department administrators). Semi-structured interviews and focus groups occurred August–November 2016.

Study investigators included healthcare providers from the US and South Africa with local hospital affiliations. The interviewers, a study investigator and a visiting US pharmacy resident, recruited front-line healthcare providers with convenience and snowball sampling, and recruited senior providers with purposive sampling. There were no participant exclusion criteria. Interviews were conducted as focus group discussions if preferred by participants. Participants were provided an informed consent document approved by the ethics committee prior to the interview and could decline participation at any time. Interviews were conducted by the interviewer in consultation rooms and offices. All interviews were conducted in English by one of the two interviewers with questions from a semi-structured interview guide and probing techniques by the interviewer. Interviews continued until thematic saturation was observed regarding barriers and facilitators for CDI treatment and management. The University of the Western Cape Research Ethics Committee granted approval for this qualitative study.

### Data analysis

Interview audio recordings were transcribed verbatim and checked for accuracy. Data analysis included coding to factors determined a priori (including key workflow steps: 1) Identification, 2) Diagnosis, 3) Treatment, and 4) Prevention) as well as inductive coding to emerging themes [7]. Two individuals from a team of three coders (LL, TE, and KP) conducted each coding phase. Paired coding with two coders per phase was performed to minimize bias. Coding schema was created to reconcile local medical terminology. Discrepancies in coding were resolved by consensus. Kappa scores were calculated to assess coding agreement at a mid-point and at the conclusion of coding. While we had initially planned to map results with the SEIPS framework, CDI management knowledge was significantly lower than expected and insufficient to frame the results in terms of tools, people, tasks, the internal environment, and the organization. Alternatively, we mapped coded themes to the workflow structure identified from the interviews.

After identifying large discrepancies in health care provider knowledge regarding CDI during the interview process, a scoring system was developed to categorize participants’ CDI knowledge from their interview responses (Table 1). The intent of the assessment was to quantify the unexpected differences. With the knowledge assessment, one knowledge point was possible from each of the following seven CDI-related components: signs and symptoms (e.g. diarrhoea), characteristics of bacteria (e.g. microbiology, virulence mechanism, disruption of normal flora, opportunistic), hand hygiene (e.g. soap and water needed to clean hands, not just alcohol), treatment (e.g. metronidazole, oral vancomycin, fecal transplant, contraindication with loperamide), contact precautions/isolation (contagious), risk factors (e.g. healthcare exposure, antibiotic use, immunocompromised by medication or illness [cancer, HIV status, CD4 count < 200] proton pump inhibitor use), and diagnosis (e.g. stool sample and testing methods, polymerase chain reaction[PCR]/toxin detection). The following responses did not receive a point allocation: 1) only stating ‘bacterial infection’ for characteristics of bacteria, 2) stating a non-specific sign and symptoms of infection or illness without stating diarrhoea, 3) stating rehydration (electrolytes) without specific antibiotic treatment name. Total knowledge score from each individual interview was further classified into four categories: ‘no knowledge’ (0–1 point), ‘limited knowledge’ (2–3 points), ‘moderate knowledge’ (4–5 points), and ‘advanced knowledge’ (6–7 points). Each CDI knowledge category was also scored across all interviewees. Researchers conducted subgroup analysis of knowledge level based on occupation and performed analysis of individual CDI assessment knowledge categories by participant and occupation. The two focus group interviews were excluded from the knowledge assessment analysis due to potential knowledge score overestimation. However, dialogue from the group interviews was included in the qualitative analysis. All analyses were conducted using NVIVO software (Version 11, QSR International).

**Table 1:** *Clostridium difficile* knowledge assessment

Criteria for *Clostridium difficile* knowledge	Points
Signs and symptoms (diarrhoea)	1
States characteristics of bacteria (any mention of: microbiology, virulence mechanism, disruption of normal flora, opportunistic)	1
Soap and water needed to clean hands, not just alcohol	1
Treatment options (any mention of: metronidazole, oral vancomycin, fecal transplant, contraindication with loperamide)	1
Contact isolation needed (or contagious)	1
Risk factors (immunocompromised, antibiotic use, proton pump inhibitors)	1
Diagnosis (stool sample, testing methods [PCR/toxins])	1
Total points	=
No knowledge = 0–1^a^ Limited knowledge = 2–3 Moderate knowledge = 4–5 Advanced knowledge = 6–7	

^a^Point allocation of 1 is considered no knowledge because there are multiple diseases associated with any one of the criteria, unless person states characteristics of bacteria

## Results

A total of 26 semi-structured interviews were conducted with healthcare providers (11 nurses, 4 pharmacists, 11 physicians) of various rankings (Table 2). In addition, two focus groups were conducted; one with seven nurses and the second with three pharmacists, resulting in 36 study participants (Table 2). Kappa scores indicated high intercoder agreement (midpoint kappa = 0.71, final kappa 0.63). The median overall CDI knowledge score from the 26 individual interviews was 3 [interquartile range 0.25, 4.75]. Subgroup median knowledge scores and an analysis of responders’ knowledge of each category are presented in Table 3. Inductive themes were coded for processes required for CDI workflow and organizational culture (beliefs and attitudes) regarding change (i.e. the ease of positive change at the organization or ‘change culture’) in order to inform future interventions. Healthcare provider responsibility and accountability for components of CDI management emerged as an organizational culture theme from the interviews. Thematic saturation of barriers and facilitators to CDI management was reached across the health care provider types (i.e. no additional themes emerged after iterative analysis of 26 interview and two focus group transcripts) [8]. CDI workflow steps are presented along with corresponding knowledge scores, barriers, and facilitators, (Section I: Workflow) and followed by organizational culture themes (Section II: Organizational Culture).

**Table 2:** Occupations and stated titles of healthcare providers interviewed

Healthcare Provider Occupation	Participants	Interviews
Nurse		
Operational managers or Assistant manager	4	4
Registered nurse or unspecified nurse	4	4
Infection Prevention and Control Nurse	2	2
Nurse Training Clinical Program Coordinator	1	1
Ward Nurses Focus Group Interview	7	1
Subtotal:	18	12
Pharmacist		
Pharmacist	4	4
Pharmacist Focus Group Interview	3	1
Subtotal:	7	5
Physician		
Head of Department	2	2
Consultant	1	1
Unspecified physician	1	1
Registrar	1	1
Medical officer	5	5
Intern	1	1
Subtotal:	11	11
Total (N)	36	28

**Table 3:** *Clostridium difficile* infection (CDI) knowledge scores overall, by healthcare provider, and each CDI knowledge category

CDI knowledge sorted by healthcare provider					
		Occupation			Overall
		Nurse (*n* = 11)	Physician (n = 11)	Pharmacist (*n* = 4)	All participants (*n* = 26)
Median Score (0–7), [1st, 3rd interquartile]		1 [0, 2.5]	5 [4, 6]	0.5 [0, 1]	3 [0.25, 4.75]
Knowledge Classification, n (%)					
No		6 (54.5)	0 (0.0)	4 (100.0)	10 (38.5)
Limited		4 (36.4)	0 (0.0)	0 (0.0)	4 (15.4)
Moderate		0 (0.0)	6 (54.5)	0 (0.0)	6 (23.1)
Advanced		1 (100.0)	5 (45.5)	0 (0.0)	6 (23.1)
Knowledge assessed in each CDI knowledge category					
Components of CDI knowledge assessment, n (%)					
1. Identification	1.1 Characteristics of bacteria	2 (18.2)	4 (36.4)	0 (0.0)	6 (23.1)
	1.2 Risk factors	3 (27.3)	10 (90.9)	0 (0.0)	13 (50.0)
	1.3 Signs and symptoms	3 (27.3)	11 (100.0)	2 (50.0)	16 (61.5)
2. Diagnosis	2.1 Diagnosis	1 (9.1)	10 (90.9)	0 (0.0)	11 (42.3)
3. Treatment	3.1 Treatment options	1 (9.1)	7 (63.6)	0 (0.0)	8 (30.8)
4. Prevention	4.1 Hand washing needed	4 (36.4)	7 (63.6)	0 (0.0)	11 (42.3)
	4.2 Need for contact isolation	4 (36.4)	8 (72.7)	0 (0.0)	12 (46.2)

### Section I: Workflow

**Fig. 1:**
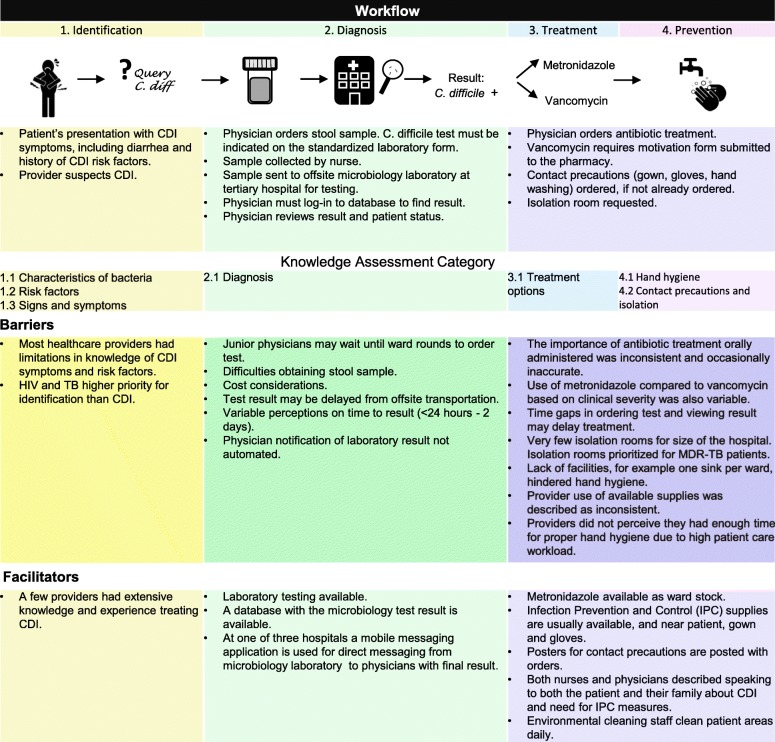
*Clostridium difficile* infection (CDI) identification, diagnosis, treatment, and prevention workflow: facilitators and barriers

Figure 1 presents workflow depicted from interview results, along with facilitators and barriers to CDI management summarized in the context of the CDI workflow, including the previously identified steps of CDI identification, diagnosis, treatment, and prevention. When CDI is suspected, a stool sample is sent to an offsite laboratory for *C. difficile* identification by PCR. Following CDI diagnosis, treatment and infection prevention and control measures are initiated. Processes were consistent between healthcare providers with knowledge of the workflow step.

#### Identification and healthcare provider knowledge

CDI identification requires knowledge of the bacteria, risk factors and clinical suspicion when patients present with CDI signs and symptoms. A major barrier to identification is low CDI knowledge. Ten interviews (6 nurses, 4 pharmacists) scored as ‘no CDI knowledge’ (Table 3). One participant candidly revealed the lack of CDI knowledge.

*“It’s actually the first time that I hear about it, to be honest”* - Pharmacist

CDI signs and symptoms were most commonly known by healthcare providers (*n* = 16, 61.5%). Thirteen (50%) participants could not describe CDI risk factors that could prompt clinical inquiry for CDI; this knowledge gap creates a potential barrier for prompt identification. Two physicians reported extensive experience with CDI in the United Kingdom. A recurrent theme from the interviews among providers was that identification for HIV and TB was prioritized over CDI. Physicians who have worked in the United Kingdom (U.K.) elaborated that the sense of urgency in South Africa for CDI was different than their previous experience due to competing attention of other prevalent disease.

*“When I was in the UK [United Kingdom] years ago… [when] the manager mentioned C. diff the staff would jump up and down and get incredibly panicky… we just don’t have that sense of urgency here… if you mention to someone in any hospital, they will go ‘Okay, what is that?’ [in cavalier tone]... however, if you tell them there is a patient with a potential XDR-TB [Extensively drug-resistant TB], then they may jump up and down. So the whole thing with C. diff it’s a reality… …a lot of people just think it’s a disease with the elderly, but we have a lot of immunocompromised patients…”* - Physician

At one hospital, CDI awareness in senior staff only increased after an outbreak in the hospital. Awareness was lower for rotating junior staff who did not experience the outbreak.

*“In terms of my junior staff, I think [CDI] ranks quite low. I think it’s got to do with the way we’ve become aware last year. We’ve had more cases making us aware that it’s highly infectious.”* - Physician

While some providers conjectured CDI to be a national problem, others did not, and no providers were aware of CDI magnitude in South Africa. Facilitating CDI identification were the senior providers with higher CDI knowledge. At one of the hospitals, an ASP was referenced as attributing to low incidence.

#### Diagnosis

After identification, to inform diagnosis, a stool sample from the patient is tested at a laboratory for *C. difficile*. While all hospitals in our study had laboratory testing available to conduct a *C. difficile* PCR test, testing occurred offsite as there was not capacity for the PCR test at the onsite laboratory. In order to test for *C. difficile*, physicians must indicate the test on a standardized laboratory form. Perception of time to result varied widely and was attributed to delays in initiating treatment. Additional barriers identified included staff difficulties obtaining stool samples due to staff shortages and non-standardized collection of laboratory samples. Laboratory test costs were occasionally cited as reasons to not test for *C. difficile*. Eleven interview participants described CDI diagnosis (42.3%, Table 3).

*“Most of the time they are not tested, because they come from the emergency, and because our emergency is so busy, then the patient is pushed up to the ward. So then only when the patient is in the ward, and then we are actually reporting the [diarrhoea] to them [post call]. And then report that the patient is having diarrhoea; then that’s the only time that they collect a stool specimen, and then after some, a couple of days, they get the results: the patient is positive. See... It could be about a week.”* - Nurse Focus Group

Other attributes identified in delaying the time to diagnosis include waiting for a physician to suggest the *C. difficile* test or until ward rounds to order it. To find results, physicians must proactively login to the database—usually from their personal mobile phones, as computer stations are not easily available. One of three hospitals uses a mobile messaging application for direct messaging from the microbiology laboratory to physicians with the goal of reducing the result notification time.

*“I think the one resource that we’ve shown very well is the communication system. I think we chose the cheapest one we could find which is WhatsApp and that does make a difference in terms of managing your patients and getting a quicker diagnosis. The thing about WhatsApp is if a patient had a positive result, it would take the doctor another 2 days to figure it out that an infection exists. We actually have an alert system that works.”* - Physician

After observing the test result, the physician informs the nursing staff if the patient has a CDI. The IPC nurses are also informed of results and may, in turn, inform the medical team. However, there is not a timely and consistent pathway for this notification, especially during post-call hours. The IPC nurse sends physicians a report including positive *C. difficile* test results on a monthly basis.

#### Treatment

Antibiotic treatment options for antibiotic-associated diarrhoea included in South African treatment guidelines at the time of the interviews were oral metronidazole initially and oral vancomycin for diarrhoea not responsive to metronidazole; vancomycin must be oral to reach the infection. Of note, the interviews were conducted prior to the revised IDSA CDI guidelines in 2018 [3]. Eight (30.8%) respondents mentioned CDI treatment options, including treatment with metronidazole and vancomycin, though the importance of antibiotic treatment administered orally was reported inconsistently and occasionally inaccurately.

A few providers also discussed the clinical use of metronidazole compared to vancomycin, including patients’ illness severity.

*“So patients who don’t respond to metronidazole would definitely be candidates for vancomycin or a metronidazole allergy.”* - Physician

Communication barriers were attributed to delays in treatment and included factors such as results being finalized while the physician was post call and drug order errors needing clarification.

Healthcare providers’ high familiarity with metronidazole and its availability on the hospital floor as ward stock facilitated its use for CDI treatment. To order vancomycin and other antibiotics on the Essential Medicine List for Hospital Level Adults, providers needed to complete a pharmacy-approved motivation form that facilitates appropriate antibiotic use. Participants reported a time gap between ordering, sending the medication chart to the pharmacy, having the medication delivered to the ward, and administrating it to the patients. Some orders might be written up and not sent to the pharmacy. For stat orders, nurses may retrieve orders from the pharmacy. The pharmacies were closed during evenings and weekends. An emergency stock of inventory is kept in the emergency center. If the needed drug is unavailable, an on-call pharmacist is called-in to prepare it. Occasionally medication was not administered and incorrectly documented as unavailable while drug was available in the emergency stock. Other reported barriers to patients receiving medications as ordered included: illegible handwriting, medication orders not including which ward an order came from, and physicians writing brand names when nurses only know the generic name. Additionally, sometimes a medication was given and not recorded; other times the patients missed doses because they were not present.

*“The problem with this is ...that sometimes the results come back, the doctor is post call. Yes, and then he will only get the feedback the next day when he is actually coming to check on his patients. So that is the delay to start”-* Nurse Focus Group

#### Prevention: Contact precautions, hand hygiene, isolation, environmental cleaning

##### Contact precautions

CDI prevention procedures include contact precautions (e.g. gown, gloves) to reduce the risk of *C. difficile* spreading to other patients. Twelve (46.2%) participants reported the need for strict contact precautions when CDI was suspected or diagnosed. Supplies and procedures for IPC (included posters displaying orders for contact precautions) were usually available but not always utilised. Supplies (including gowns, gloves, masks, and hand sanitizer) were available in close proximity to a patient once contact precautions were ordered. Staff education and timely notification of need for infection control were the most common barriers to IPC measures. Pressure from patient bed shortages can lead to patients being placed near each other. Contact precautions with the first suspicion of CDI was described at one of the hospitals.

*“...any patient with diarrhoea is placed with contact precaution; until we know if they have been exposed to any antibiotics, we put them as high risk.”* - Physician

At the three hospitals, the ward nurse in charge will enforce contact precautions with the nurses and the attending/consulting physicians will enforce junior physicians’ contact precautions. The IPC team also enforces IPC practices. Both physicians and nurses inform patients about contact precautions; patients are told to inform their family members. While senior physicians reported informing patients of the need for IPC in the CDI setting, nurses considered themselves more approachable than the physicians and took a primary role in communicating with patients. One junior physician admitted his/her peers’ shortfalling.

*“I think that from all of it, that is where the biggest failing comes in—that we often don’t tell patients enough of the stuff. So, I would like to think that once it’s done there is a proper [communication] about the patient having things that can be transmitted, with words that they can understand and the importance of them not going around and touching lots of things and letting them know the reasons for gloving up and putting on gowns and stuff for their own peace of mind…It’s apathy from the medical staff we forget to do these things...”* - Physician

##### Hand hygiene

Facilitators and barriers to hand hygiene were related to the treatment of patients with CDI and additional infections. Hand hygiene practices for patients with CDI should include hand washing with soap and water to remove *C. difficile* spores that are not killed by alcohol hand sanitizers. Supplies, including paper towels, soap, and hand sanitizer, were frequently available but not always utilised. Some stated that insufficient supplies were a barrier; others said that supplies were always available. Eleven (42.3%) participants acknowledged the importance of washing hands with soap and water when treating patients with CDI (Table 3).

*“...have to use soap and water, we take [the] de-germ [alcohol based hand sanitizer] away from bedside so they are forced to use soap and water.”* - Physician

Some perceptions regarding this important hand hygiene practice were inaccurate.

*“I would not say a normal hand soap is better for* C. diff*, I would say something alcohol based.” -* Physician

Staffing shortages and high workload were described as reasons for inconsistent hand hygiene practices.

*“Can I tell you, all over the basins is that sign [WHO’s “5 Moments of Hand Hygiene”]… but we don’t practice it...We don’t follow five moments of Hand Hygiene. We follow it when we go home... You can’t afford to take that 5 min.”* – Nurse Focus Group

Participants described hand hygiene events (e.g. ultraviolent light, blue soap) in their hospitals that encouraged effective hand hygiene. Many stated that overcrowding and lack of facilities (e.g. one sink per ward) hindered hand hygiene as well as: the high ratio of patients to nurses, education limitations, and sometimes-empty alcohol and/or soap dispensers.

##### Isolation

Infrastructure limitations were a major barrier to IPC, often preventing CDI patients from allocation to an isolation room. Isolation room availability ranged from two to four rooms. Isolation rooms were specifically prioritized for multidrug resistant tuberculosis (MDR-TB) patients, who may occupy the room for a month. CDI is viewed as a lower priority for isolation rooms.

*“The fact that we have got a lot of immunocompromised patients in terms of our HIV rates and TB rates, a lot of our patients are at risk due to the use of antibiotics. In the UK we used to see a lot of elderly patients, but here you have got a different spectrum of patients, so C. diff is a huge risk… I think everyone focuses on MDR and very few people actually focus on C. diff … C. diff is not something that is high on the radar.”* - Physician

Challenges for IPC included patient education regarding IPC, especially patients leaving isolation, walking around the hospital, and using shared bathrooms.

*“The big problem that we have in our wards is a lack of isolation facilities. For an entire hospital, we’ve got only four isolation rooms [that] do not include isolation bathrooms. So a C. diff patient would have to use the same toilet as other patients.”* - Physician

Both nurses and physicians described speaking to patients and their family members about isolation. An elevated desire from patients to understand their condition was expressed when patients were moved to an isolation room.

*“Sometimes you’ll find the patient doesn’t know what is going on, but when you move them into an isolation room then they want to know why.”* - Nurse

##### Environmental cleaning

The ward managers inform cleaning staff verbally about room cleaning needs. Under supervision, the cleaners complete a written checklist for the bathrooms and patient rooms. Cleaning is sometimes rushed due to high bed demand, and the staff nurses will help.

*“It’s just that we are busy so the beds are always in demand so sometimes there is no opportunity for cleaning because everything is rush, rush, rush, rush. When the patient is waiting on discharge, others are waiting for that bed so we don’t have the opportunity to do the spring cleaning of the unit. We aren’t always able to do it in a calm environment.”* – Nurse

### Section II: Organizational Culture

Themes related to organizational culture (beliefs and attitudes) and how leadership and administration respond to new ideas, specifically ‘change culture’, were analyzed in order to inform future interventions. Through this coding an additional organizational culture theme emerged related to healthcare provider responsibility and accountability.

#### Change culture: how leadership and administration respond to new ideas

The majority of respondents described leadership as being supportive of new ideas. Some respondents did not feel leadership was supportive of bottom-up ideas; others believed that ideas with evidence of positive impact would be supported. A few respondents noted a barrier to change related more to nursing staff and junior physician turnover than to administrative support. Progressive change is difficult when the same education concepts are repeated with rotating healthcare providers; institutional memory regarding CDI and CDI management was lost.

“*Implementing change and practical change are very different, so we are able to change our practice so we can make lots of suggestions... but the difficulty comes in that our staff [is a] rotating staff*.” - Physician

A nurse new to a leadership position anticipated facing challenges in changing long-standing practices.

*“The people above me, the specialist physicians or consultants, are quite open to change. If you can show clearly that an idea is going to work, the department is open to change and improving things. As you get higher up the leadership chain, it becomes more difficult to introduce change. I do find that on the face of it, the managers seem to be okay and accepting and are happy to listen.”* - Physician

#### Responsibility and accountability

While the interviewees described achievements of and challenges for patients and healthcare providers following IPC precautions, low adherence emerged as a compelling theme—sometimes in the context of IPC in general and for the treatment of TB, particularly when participants had limited CDI knowledge. Perception of the threats from infectious diseases and IPC prioritization also appear to be barriers to adherence when supplies are available. Accountability structures are not in place to properly encourage providers to remain knowledgeable about guidelines nor enforce IPC precautions.

*“It seems we have many awareness days… we had spike last year, 2 years ago… we have had quite a few staff members contracted tuberculosis… people only get aware if their buddy gets it… It makes it real*.” - Physician

*“Just to get the doctors to wear gloves—that for me is another thing where I can just say… like, ‘Why are you not wearing gloves?’ or, just tell them ‘Your patient has TB. Can you put on your mask please?’ ...together with the hand washing, and at the end of the day, it is part of the IPC principles to have full personal protection equipment available in the unit, but there’s hand sanitizers, soap, and water, available in the unit, so no one has an excuse.”* - Physician

Informal structures for peer accountability were discussed as a helpful strategy from two interviews. First, accountability for hand hygiene occurred on the ASP ward round at one hospital. Second, an Operational Manager in the Operating Room (Theater) described nursing and cleaning staff who speak up about needs and follow cleaning expectations.

*“The cleaning staff and the nursing staff is quite well informed as to what is supposed to happen, because sometimes they can tell you. ‘Sister, this was not done yet; You can’t really put your patient here’… Those are the people that I work with... that I come across, that will tell me. Doesn’t matter if you are the cleaner, you can tell me, ‘Sister, it’s not ready yet.’ You understand. It’s that relationship that we have [of a] multidisciplinary team, to do what is expected of us.”* – Nurse

## Discussion

### Principal findings

This is the first qualitative study of CDI in Sub-Saharan Africa, and the results provide novel insight into CDI treatment and workflow in a limited resource setting. The context of CDI in Africa is especially important to consider given the high HIV and tuberculosis prevalence and high risk of *C. difficile* associated mortality in this population. This study reveals significant barriers and facilitators to CDI treatment in public district (secondary) level South African hospitals. Major barriers included knowledge gaps in CDI management, especially regarding awareness of the infection, transmission, treatment, and IPC practices among health care providers. Physician CDI knowledge was higher than nurse and pharmacist knowledge. The results reveal opportunities for healthcare provider education related to CDI. Our study affirms that healthcare providers have an awareness of evidence-based IPC precautions but barriers to following them include perceptions of priority and time availability.

### Implications: perceptions and knowledge

Based on quantitative results from the overall CDI knowledge assessment, participants had limited CDI knowledge. Gaps in CDI knowledge may delay clinical suspicion and all workflow steps in CDI identification, diagnosis, treatment and prevention. While physicians scored higher, some physicians were less confident regarding when to order the *C. difficile* test resulting in delayed diagnosis. Physicians with high CDI knowledge noted an urgency surrounding CDI not observed in junior physicians and other healthcare providers.. This, together with a high risk of mortality in patients with positive *C. difficile* test results, underscores an urgent need for education and intervention tailored to relevant aspects of healthcare providers’ job responsibilities.

Overall, participants scored well in areas of identifying CDI risk factors, signs, and symptoms. However, improvement is needed in terms of educating healthcare professionals in South Africa about other aspects of CDI. In the occupation subgroup analysis, nurses and pharmacists appear to be less knowledgeable about CDI characteristics, with response rates of 50% or less in all the knowledge assessment categories. The identified areas for potential development relevant to nurses and pharmacists are: CDI patients’ need for contact isolation, the importance of hand washing instead of using alcohol gel in preventing the spread of CDI, and CDI treatment options. Nurses can also be educated to suspect CDI when monitoring bowel movements.

This study reveals a more complicated process for obtaining and administering vancomycin compared to metronidazole that may be hindering healthcare providers’ use of vancomycin. In an epidemiology, treatment, and outcomes study in the same setting, vancomycin was rarely ordered (2%) as initial CDI treatment [4]. One strategy is to incorporate treatment options for CDI into pharmacist education and teach pharmacists what to look for on physician-submitted motivation forms. Pharmacist education about treatment options is especially important considering the role pharmacists have in the approval process for vancomycin use. The healthcare team should be educated on the clinical use of vancomycin for CDI with an emphasis on timely preparation and delivery.

### How results relate to other studies

Our study affirms current literature’s described need for improved CDI identification in settings with extensive CDI experience. Despite a history of substantial CDI outbreaks in Europe, a study identified persistent underdiagnoses of CDI when all diarrhoea samples were tested at 482 hospitals across 20 European countries; 23% of *C. difficile* positive results were not identified at the local hospital. Authors attributed the underdiagnoses to a lack of clinical suspicion and suboptimal laboratory diagnostic methods [9]. Meanwhile, in the US, a regulatory climate that reduces hospital reimbursement for patients who develop hospital-acquired infections is driving efforts to refine testing protocols to avoid *C. difficile* over testing and inappropriate diagnosis [10]. These studies emphasize the importance of appropriate testing for diagnosis.

A global review of CDI guidelines found antimicrobial stewardship (ASPs) to be universally recognized as an essential evidence-based component of CDI IPC [5]. Continued development of interdisciplinary ASPs in limited resource settings is necessary to facilitate effective CDI management and IPC measures.

One barrier to hand hygiene identified in this study was the perception that there is insufficient time available for thorough hand cleaning. Indeed, in a study conducted in the US about healthcare providers’ compliance with IPC practices for patients with CDI, full compliance was very low and time-consuming with a mean time for full compliance greater than 5 min for patients in single isolation rooms [11]. Patient care workload continues to be a barrier to full compliance with CDI contact precautions in high resource settings [12]. Therefore, improving full compliance of IPC practices in limited resource settings will require both a workload adjustment to allow more time per patient and education on the importance of CDI-related IPC practices.

Significant challenges for the implementation of IPC programs and practices exist in low and limited resource settings, including infrastructural constraints with a limited number of isolation rooms and variable staff compliance with hand hygiene practices. A similar qualitative study in India found perceived workload and nursing staff turnover to be barriers to infection control [13]. This relates to our study’s previously referenced finding that perceived workload hindered infection control practices, especially regarding hand hygiene. Our respondents reported high turnover of both nursing staff and junior physicians as barriers to implementing change. The secondary hospitals included in our study did not have an IPC team as developed as the one in the tertiary hospital in India. The study in India also found participants reporting the availability of IPC supplies but experiencing challenges with compliance, while an international study of healthcare settings representing 30 countries identified inadequate supplies as a barrier to infection control of multidrug resistant organisms in some high and middle income countries [13, 14].

### Limitations

As a qualitative study, the results are not generalizable to a larger population but may be transferable to similar settings. Visiting researchers’ presence conducting the interviews may have affected responses; stated practices are not necessarily the reality of practice. While all interviews were conducted in English, English was a second language for some participants. This may have limited the respondents’ understanding of some questions and ability to articulate responses. Furthermore, we may have underestimated facilitators to CDI management in an attempt to identify improvement opportunities. Our analysis was not a systematic audit of workflow and practices, and some inaccuracies may exist. To mitigate bias, multiple researchers of the study team reviewed the results. Finally, as we developed the knowledge assessment after the interviews were completed, the assessment is not yet validated and results are limited. Our knowledge assessment measured breadth of CDI knowledge and not depth. For example, some providers gave detailed explanations for some of the knowledge components, such as advantages of different testing protocols, yet these explanations were still only assigned one point for that component.

## Conclusions

Our analysis provides a detailed description of the facilitators and barriers to CDI workflow, including the need for increased healthcare provider knowledge of CDI management. Interventions should increase CDI knowledge and utilization of the available systems and supplies by addressing the identified barriers and championing the identified facilitators. Increasing CDI knowledge alone is unlikely to be effective without addressing the need to create a sense of urgency around CDI and appropriate IPC practices. The results provide context for technical intervention and implementation strategies in low-resource public healthcare settings. This study serves as a baseline and supplements quantitative CDI patient data from ongoing CDI research including provider education and a clinical intervention to improve CDI quality of care in South Africa. The results of this workflow and provider knowledge analysis identify areas of need and are useful to design interventions to improve the quality of care for CDI patients in this population and similar limited resource settings.

## Abbreviations

ASP: Antimicrobial stewardship programs
CDI: *Clostridium difficile* infection
IPC: Infection prevention and control
MDR(-TB): Multidrug resistant(−tuberculosis)
PCR: Polymerase chain reaction
SEIPS: Systems Engineering Initiative for Patient Safety
TB: Tuberculosis
UK: United Kingdom
US: United States
XDR-TB: Extensively drug-resistant TB
